# The *Arabidopsis thaliana* SERK1 Kinase Domain Spontaneously Refolds to an Active State *In Vitro*

**DOI:** 10.1371/journal.pone.0050907

**Published:** 2012-12-07

**Authors:** Marije aan den Toorn, Mieke M. E. Huijbers, Sacco C. de Vries, Carlo P. M. van Mierlo

**Affiliations:** 1 Laboratory of Biochemistry, Wageningen University, Wageningen, The Netherlands; 2 Centre for Biosystems Genomics, Wageningen, The Netherlands; University of South Florida College of Medicine, United States of America

## Abstract

Auto-phosphorylating kinase activity of plant leucine-rich-repeat receptor-like kinases (LRR-RLK's) needs to be under tight negative control to avoid unscheduled activation. One way to achieve this would be to keep these kinase domains as intrinsically disordered protein (IDP) during synthesis and transport to its final location. Subsequent folding, which may depend on chaperone activity or presence of interaction partners, is then required for full activation of the kinase domain. Bacterially produced SERK1 kinase domain was previously shown to be an active Ser/Thr kinase. SERK1 is predicted to contain a disordered region in kinase domains X and XI. Here, we show that loss of structure of the SERK1 kinase domain during unfolding is intimately linked to loss of activity. Phosphorylation of the SERK1 kinase domain neither changes its structure nor its stability. Unfolded SERK1 kinase has no autophosphorylation activity and upon removal of denaturant about one half of the protein population spontaneously refolds to an active protein *in vitro*. Thus, neither chaperones nor interaction partners are required during folding of this protein to its catalytically active state.

## Introduction

Plant Receptor-like Kinases (RLK's) belong to a large gene family [1] involved in perceiving developmental cues and environmental changes. Activation of receptor-like kinases is thought to occur upon ligand induced complex formation, followed by activation of downstream associated proteins [2]. In inactive receptors, activity of the kinase domain is often regulated by several methods of control, such as (release of) inhibitors [3], dimerization [4], activation segment phosphorylation [5], and changes in sub-cellular localization [6]. Although a huge variety in modes of regulation exist among RLK's, activation of protein kinases is usually coupled to conformational changes in protein structure [7].

**Figure 1:**
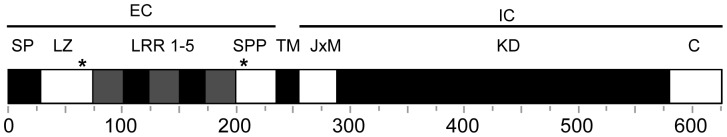
Domain architecture of SERK1. SP = signal peptide, LZ = leucine zipper, LRR = leucine-rich-repeats, SPP = serine-proline rich domain, TM = single pass transmembrane domain, JxM = juxtamembrane domain, KD = kinase domain, C = C-terminal tail, EC = extracellular domain, and IC = intracellular domain. Two cysteine pairs in the extracellular domain are depicted with *, and numbers below the graph indicate amino acid residues.

Here, we focus on an RLK from *Arabidopsis thaliana*, the Somatic Embryogenesis Receptor-like Kinase 1 (SERK1). SERK1 is part of a protein family consisting of five highly conserved leucine-rich-repeat receptor-like kinases that function in plant signalling pathways [8], [9], [10]. SERK proteins are thought to be co-receptors, which are required for transmission of signal [11] but are not able to bind ligand themselves [12]. The SERK1 domain overview is shown in Fig. 1.

Previous studies show that the kinase domain of SERK1 (SERK1-KD) is active *in vitro* [13], and has the highest autophosphorylation activity of the SERK protein family [14]. SERK1 has been implicated to function in somatic embryogenesis, male sporogenesis and brassinosteroid (BR) signalling in *Arabidopsis thaliana* [8], [9], [15], [16]. The three-dimensional structure of the kinase domain of a close paralog of SERK1, BAK1 (also called SERK3) in complex with AvrPtoB has been elucidated [17]. This structure is similar to the one adopted by several tyrosine kinases. BAK1 seems to belong to the IRAK family of kinases, because it contains a tyrosine as “gatekeeper” residue [17]. The “gatekeeper” controls access to a hydrophobic pocket behind the ATP-binding site, and probably plays a role in selectivity of a kinase with regard to small-molecule, ATP-competitive inhibitors [18]. The tyrosine gatekeeper identified in BAK1 (i.e., Y363) is conserved in the SERK1 sequence (i.e., Y376), indicating that SERK1-KD is a member of the IRAK family of protein kinases.

SERK1 functions in an oligomeric complex with other RLK's such as BRI1, the main ligand binding receptor for BRs [19], and transphosphorylation occurs between these receptors [14], [20]. For the BR signalling pathway, SERK co-receptors are required for BRI1-mediated signal transduction, and whole seedlings lacking three of five family members completely lack dephosphorylation of the transcriptional regulator BES1 as a read-out of BR signalling [11]. Interaction between BRI1 and SERK proteins possibly involves the extracellular domains of both proteins, and is induced upon ligand binding to the extracellular domain of BRI1 [21]. Fluorescence Correlation Spectroscopy (FCS) shows that in protoplasts that have not been treated with BRs, about 15% of SERK1 proteins exist as dimers [22]. SERK1 interacts with PP2C type phosphatase KAPP, CDC 48 and 14-3-3 protein GF14λ [23], [24].

Since RLK activation is often the first response to a signal, RLK's need to be kept in the non-responsive or “off”-state in absence of activating ligands. One way to achieve this would be to keep these RLK's disordered during synthesis and transport to their final location. Many proteins are intrinsically disordered and fold, in whole or in part, upon binding to their physiological targets [25], [26], [27]. Protein phosphorylation often occurs within intrinsically disordered protein regions [28]. Even in protein complexes significant regions of intrinsic disorder can exist [29]. Especially in eukaryotes many proteins seem to contain unstructured regions of significant size (>50 residues) or are completely disordered [27], [30], [31]. IDP's function in transcription, translation, signal transduction and protein assembly [32], [33]. These unstructured proteins do not fold until their activity is required and folding is triggered by, for instance, binding of a substrate or a dimerization partner, or a change in subcellular localization [25]. For the *Arabidopsis thaliana* proteome, it has been predicted that 29% of its proteins contain intrinsically disordered regions, and 8% of its proteome consists of completely disordered proteins [34].

An example of IDP's in plants is the GRAS-protein family of transcriptional regulators [35]. This protein family functions in several pathways in plant development and signal transduction, including the brassinosteroid pathway [35]. Disordered regions of these proteins are thought to enable a particular protein to interact with different partners and as a result it can function in different signalling pathways [36]. Also, plant specific plasma membrane-located remorin proteins, part of signal transduction cascades, have intrinsically disordered regions [37]. For both GRAS proteins and remorins, phosphorylation is an important component during their modulation [35], [37].

In this study we show that use of a disorder analysis computer program predicts that the kinase domain of SERK1 (SERK1-KD) possibly contains a disordered region. We report experimental data of folding, stability and folding-induced phosphorylation activity of SERK1-KD *in vitro*. These data show that SERK1-KD predominantly is a structured protein at ambient condition. Loss of this structure is intimately linked to loss of activity of SERK1-KD. *In vitro*, unfolded SERK1-KD spontaneously refolds to an active state in absence of transphosphorylating partners or chaperones.

## Materials and Methods

### Disorder analysis

Kinase domains and C-terminal tails of SERK1 (AT1G71830, residues 295–625), SERK3/BAK1 (AT4G33430, residues 282–615), FLS2 (AT5G46330, residues 863–1173), BRI1 (AT4G39400, residues 877–1196), MPK3 (AT3G45640, residues 30–370), EFR (AT5G20480 residues 705–1031) and BSK1 (At4G35230, residues 66–413) were defined by sequence alignment using Clustal Omega (version 1.1.0) from the EBI web server (www.ebi.ac.uk/Tools/msa/clustalo/). Potential presence of disordered regions within these protein sequences was analysed using PONDR® VL-XT (www.pondr.com) [38], [39], [40], VSL2B (www.dabi.temple.edu/disprot/) [41], [42] and IUPred (iupred.enzim.hu/) [43].

### Protein purification and sample preparation

SERK1-KD (residues 285–625) was cloned into the pET151 bacterial expression vector and contains an N-terminal His-tag (Invitrogen). Primers used for cloning were CAC CGG ACA GCT CAA GAG GTT TTC T and TTA CCT TGG ACC AGA TAA CTC AAC GGC. *Escherichia coli* BL21* (Invitrogen) cells containing plasmid were grown in LB at 37**°**C until OD_600_ = 0.8, after which the culture was cooled to 20**°**C and protein production was started by addition of IPTG. After 20 h cells were harvested. Cells were broken using a French Press. The protein was purified by running cell free extract over a HIS-pure cobalt column (Pierce Biotech, bed volume of 8 ml). Subsequently, protein was further purified through use of a SourceQ-15 column. Final purification was achieved by analytical gel filtration on a Superdex 75 10/300 GL (see Fig. S1). Purification of SERK1-KD was followed by standard sodium dodecyl sulfate polyacrylamide gel electrophoresis (SDS-PAGE). Purified protein was suspended in 20 mM Hepes, 150 mM NaCl, 10 mM MgCl_2_, 1 mM DTT, pH 7.4. All measurements were performed in this buffer, unless stated otherwise. A total of about 5 mg of pure fusion protein was obtained from 6 L of culture. Figure S2 shows that SERK1-KD produced in *E. coli* has the ability to autophosphorylate itself, and to transphosphorylate artificial substrate casein (Sigma).

Phosphorylated SERK1-KD was obtained by incubating 24 µM SERK1-KD with 3 mM ATP for 45 min at 30**°**C. ATP was subsequently removed using a P-10 desalting column (Amersham Biosciences). For determination of phosphorylation status, SERK1-KD after autophosphorylation was subjected to immunoblotting with anti-Phosphoserine, anti-Phosphothreonine and anti-Phosphotyrosine antibodies (BD biosciences).

### Fluorescence spectroscopy

Fluorescence was measured using a Varian Cary Eclipse Fluorescence Spectrophotometer.

For denaturant induced unfolding, 0.24 µM SERK1-KD was incubated for 6 h at room temperature at various concentrations of urea, after which fluorescence spectra were recorded. For refolding, 12 µM SERK1-KD was first unfolded in 4.5 M urea for a period of 6 h. Subsequently, the sample was diluted 50 fold with buffer containing no urea and refolding took place during a period of 30 min at room temperature. Fluorescence emission was acquired from 290 to 600 nm, using an excitation wavelength of 280 nm with emission and excitation slits set to 10 nm. For each measurement, 5 scans were averaged and every nm a data point was collected. All spectra were recorded at 20**°**C in 10 mm quartz cuvettes.

Fluorescence emission of SERK1-KD was also recorded during its temperature induced unfolding, which was achieved by increasing temperature from 15**°**C to 70**°**C at a rate of 1**°**C/min. Data points were collected every 0.5**°**C. Protein concentration of both phosphorylated and native SERK1-KD was approximately 0.24 µM. Excitation was at 280 nm, and fluorescence emission was recorded at 340 nm. Emission and excitation slits were set to 10 nm. The thermal midpoint of unfolding as reported by fluorescence spectroscopy was determined by using a two state model of unfolding [44].

### Circular dichroism (CD)

Far-UV CD data were acquired on a Jasco J-715 Spectropolarimeter. Spectra were recorded at 20**°**C using 1 mm quartz cuvettes. All spectra were corrected by subtracting spectra of corresponding blank solutions. Buffer is 10 mM NaPi, pH 7.4, unless stated otherwise.

For acquisition of far-UV CD spectra of native and phosphorylated SERK1-KD, samples had a protein concentration of 5 µM and 5.5 µM respectively. Spectra were obtained by averaging 20 wavelength scans acquired from 185 to 260 nm. A data point was collected every 0.2 nm.

For acquisition of far-UV CD spectra of native SERK1-KD in 0.45 M urea, 1.5 µM protein was incubated with 0.45 M urea. For spectra of unfolded SERK1-KD, 2.4 µM protein was incubated with 4.5 M urea. Both samples were kept at room temperature for 6 h before measurements commenced. For spectra of refolded SERK1-KD, native SERK1-KD at a concentration of 17 µM was first unfolded by incubation with 4.5 M urea for 6 h at room temperature. Subsequently, the sample was diluted 10 fold with buffer containing no urea and refolding took place during a period of 30 min at room temperature. Far-UV CD spectra were obtained by averaging 20 wavelength scans acquired from 210 to 260 nm. Below 210 nm the presence of urea causes considerable scatter of the CD signal. A data point was collected every 0.2 nm.

Thermal unfolding of 5 µM native and 5.5 µM phosphorylated SERK1-KD was followed at 210 nm. Temperature was increased from 15**°**C to 80**°**C at a rate of 1**°**C/min and a data point was collected every 0.5**°**C. The thermal midpoint of unfolding as reported by far-UV CD spectroscopy was determined by using a two state model of unfolding [44].

### Kinase phosphorylation assay

For each measurement of SERK1-KD autophosphorylation, 0.5 µM SERK1-KD was incubated at a particular concentration of urea (ranging from 0 M to 3 M urea). All samples were kept at room temperature for 6 h before the assay was started. For refolding, 8 µM SERK1-KD was first unfolded in 3 M urea for 6 h at room temperature. Subsequently this sample was diluted with buffer containing no urea to a final concentration of 0.18 M urea and 0.5 µM SERK1-KD. Refolding took place during a period of 30 min at room temperature.

To measure kinase phosphorylation activity, cold ATP and 500 µCi ^32^P-γATP (Perkin Elmer) was added to the samples. Final concentration of ATP in each sample was 50 µM. Phosphorylation took place for 30 min at 30**°**C and was stopped by addition of SDS-loading buffer. Samples were subsequently boiled for 3 min at 100**°**C and separated by SDS-PAGE. Incorporated radioactive phosphate was determined using a PhosphorImager. Relative intensities of radioactive bands were analysed using ImageJ software (ImageJ 1.45 s). Background intensity was subtracted and intensity at 0 M urea was set to 100%.

## Results

### SERK1-KD potentially contains disordered segments

**Figure 2:**
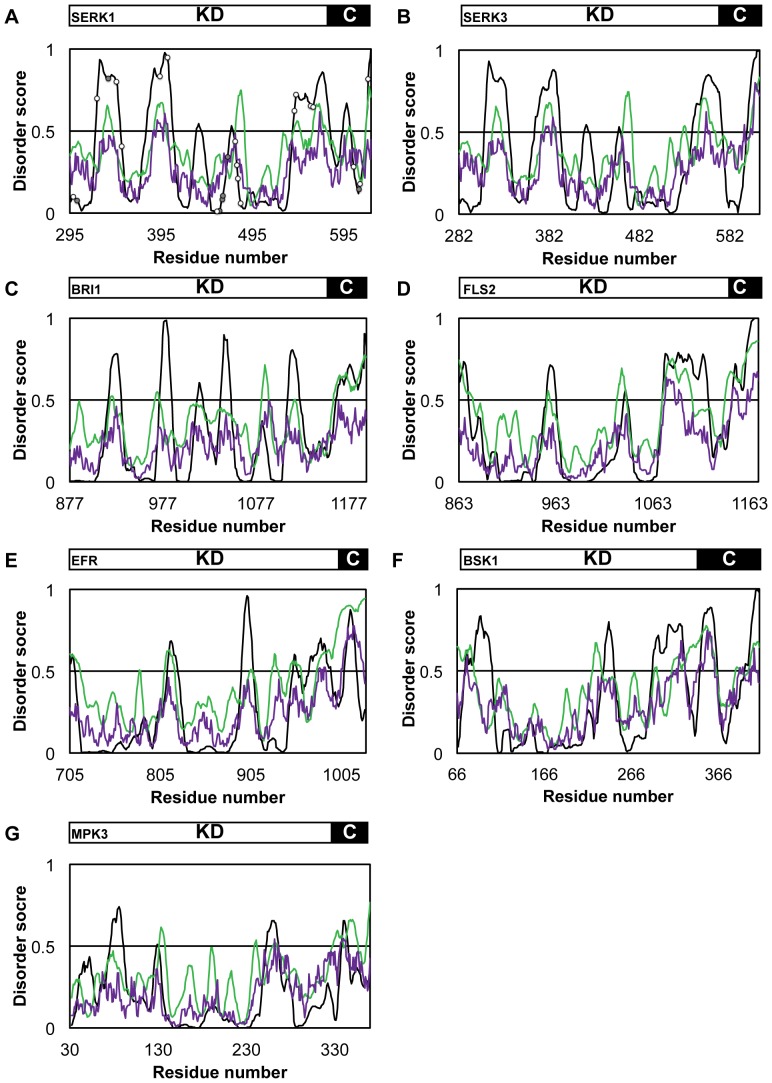
Predicted disorder within plant kinases using various disorder analyses programs. A disorder score lower than 0.5 indicates that a region is predicted to be ordered, whereas scores above 0.5 predict disordered protein sequences. Schematic above the graph indicates which part of the primary sequence belongs to the catalytic kinase domain (KD) or to the C-terminal tail (C). Disorder prediction of PONDR® VL-XT is depicted in black, VSL2B disorder analysis is depicted in green and IUPred disorder analysis in purple. (**A**) Disorder prediction for SERK1-KD. White dots indicate phosphorylation sites identified *in vitro*, grey dots indicate phosphorylation sites identified *in planta* [14]. Disorder profiles are also shown for kinase domains of (**B**) BAK1 (SERK3), a close paralog of SERK1, (**C**) BRI1, (**D**) FLS2 and (**E**) EFR, three main ligand binding plant RLK's, and for (**F**) BSK1 and (**G**) MPK3, two cytoplasmic kinases.

To identify possible regions of disorder within SERK1-KD, we use disorder predictors PONDR® VL-XT, VSL2B and IUPred. SERK1-KD consists of the catalytic kinase domain (residues 295–581) and a C-terminal region (the C-terminal tail) of 45 amino acids. Figure 2a shows that although SERK1-KD seems to be largely structured, several short segments of its primary sequence are potentially disordered, as suggested by PONDR® VL-XT. These segments include its final C-terminal residues and a segment of more than 40 amino acids in the C-terminal part of the catalytic domain of SERK1-KD (residues 540–580).

As a comparison, disorder predicted for several other plant kinases is also analysed. Sequence alignment identified the catalytic kinase domain and the C-terminal tail of these proteins. BAK1 (SERK3) is a close paralog of SERK1. Its disorder profile (Fig. 2b) strongly resembles the one of SERK1.

BRI1, FLS2 and EFR are the main ligand binding LRR-RLK's for brassinosteroid, flagellin and EF-Tu respectively [19], [45], [46]. All three receptors potentially contain a disordered C-terminal tail (Figs. 2c–e). PONDR® VL-XT suggests that only FLS2 has a disordered segment of more than 40 amino acids in the C-terminal part of its catalytic domain, just as SERK1.

BSK1 and MPK3 are cytoplasmic kinases functional in brassinosteroid and flagellin signalling, respectively [47], [48]. Both proteins apparently have no disordered segments of more than 40 amino acids (Figs. 2f,g). BSK1 does contain several short segments of predicted disorder, including disorder of its final C-terminal residues, whereas MPK3 is much more ordered (Figs. 2f,g).

Protein phosphorylation is thought to occur predominantly within disordered regions of a protein [28]. Figure 2a shows that SERK1-KD contains many phosphorylation sites [14], however, no bias towards disordered regions exists.

### Phosphorylation hardly affects the structure of SERK1-KD

Phosphorylation of the activation loop is a major event in kinase activation and involves extensive structural movements [7]. SERK1-KD purified from *E. coli* is referred to here as native SERK1-KD. To assess whether phosphorylation of the kinase domain of SERK1 alters protein structure, and to obtain insight into potential control mechanisms of kinase activity *in planta*, we acquired far-UV CD spectra of native SERK1-KD and of *in vitro* autophosphorylated SERK1-KD. Phosphorylation status of SERK1-KD is assessed with antiphospho Ser/Thr/Tyr-antibodies. Native SERK1-KD turns out to be only marginally phosphorylated compared to *in vitro* autophosphorylated proteins (see Fig. S3).

**Figure 3:**
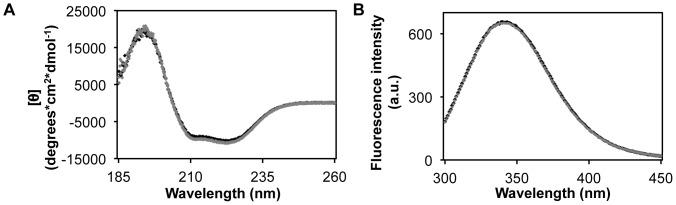
Phosphorylation hardly affects the structure of SERK1-KD. (**A**) Far-UV CD spectra of native SERK1-KD (black) and phosphorylated SERK1-KD (grey) at 15°C. (**B**) Fluorescence emission spectra of 22 µM native SERK1-KD (black) and of 25 µM of phosphorylated SERK1-KD (gray), both acquired at 20°C. Overlay of both spectra is obtained upon correcting for protein concentration difference.

Far-UV CD (Fig. 3a) shows that SERK1-KD purified from *E. coli* is well structured, because it has a spectrum that is typical for α+β proteins and is similar to the far-UV CD spectrum of BAK1-KD [17]. *In vitro* autophosphorylation of SERK1-KD is maximal after 35 min of incubation [13]. After 45 min of autophosphorylation activity, little change in secondary structure of SERK1-KD is observed (Fig. 3a). SERK1-KD has five tryptophan residues, which are distributed across the protein. Fluorescence emission reports the local microenvironment of tryptophan. Figure 3b shows that phosphorylation does not result in detectable change in tertiary structure of the protein, as is shown by the unaltered fluorescence emission maximum at 340 nm and the constant shape of the fluorescence emission spectrum. In interpreting these data, it is important to note that after auto-phosphorylation both phosphorylated and non-phosphorylated residues are present for most SERK1 phospho-sites [14].

### Phosphorylation hardly alters the stability of SERK1-KD against unfolding

**Figure 4:**
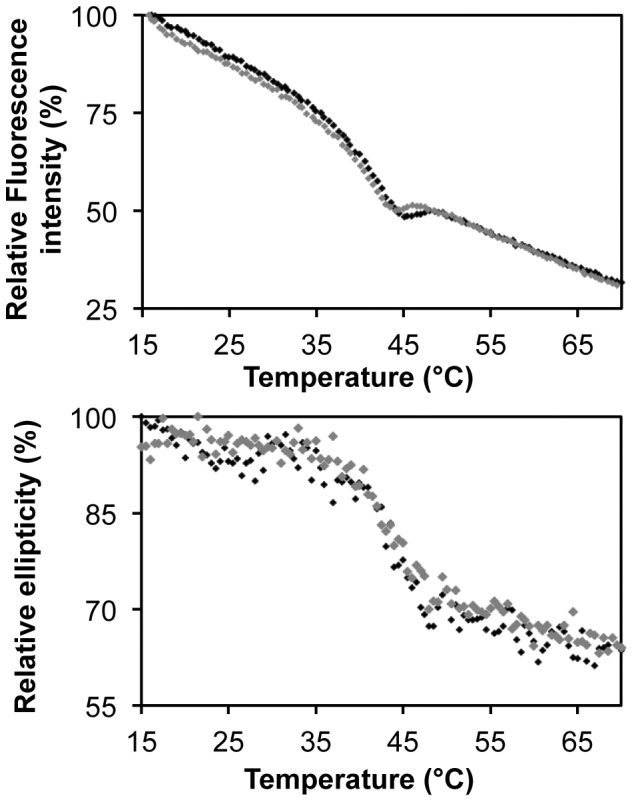
Effect of phosphorylation on stability of SERK1-KD against thermal unfolding. Top: Thermal unfolding monitored by the change in fluorescence emission at 340 nm, upon excitation at 280 nm. Bottom: Thermal unfolding monitored by the change in ellipticity at 210 nm. Data of native SERK1-KD are shown in black, and data of phosphorylated SERK1-KD are shown in grey.

Although phosphorylation *in vitro* does not affect the structure of SERK1-KD, phosphorylation might alter SERK1 kinase stability against unfolding and thereby influence its function *in planta*. To probe this phenomenon, thermal unfolding of native and autophosphorylated SERK1-KD is followed by fluorescence spectroscopy (Fig. 4a). This method probes the tertiary microenvironment of tryptophan and tyrosine residues within a protein, and loss of fluorescence intensity at 340 nm upon excitation at 280 nm indicates loss of tertiary structure. In addition, far UV-CD spectroscopy is used to probe loss of secondary structure upon thermal unfolding of SERK1-KD (Fig. 4b). Fluorescence emission of SERK1-KD drops in the 15 to 35°C temperature range (Fig. 4a), whereas ellipticity is hardly altered in the native baseline part of the thermal unfolding curve, as indicated by far-UV CD (Fig. 4b). Global protein unfolding occurs in the 35 to 45°C temperature range, as cooperative unfolding transitions in both far-UV CD and fluorescence spectroscopy data show. Fluorescence of free tryptophan is rather sensitive to temperature, with fluorescence decreasing upon increasing temperature [49]. In case of SERK1-KD, sensitivity of tryptophan fluorescence to temperature results in the slope of the native baseline of its thermal unfolding curve being negative. Possibly, increased conformational flexibility of native protein upon increasing temperature may cause this decrease in fluorescence.

Figure 4 shows that autophosphorylation hardly affects the stability of SERK1-KD against thermal unfolding. Midpoints of unfolding of native and phosphorylated SERK1-KD as determined by fluorescence spectroscopy are 40.2**°**C and 39.4**°**C, respectively. The corresponding midpoints of unfolding as determined by far-UV CD are 42.7**°**C and 43.7**°**C, respectively. Upon cooling, thermally unfolded SERK1-KD protein is not able to regain its native structure or its catalytic activity (see Fig. S4 and data not shown).

### Unfolding of SERK1-KD is intimately linked to loss of its phosphorylation activity

Unfolding of SERK1-KD is also investigated by adding urea and subsequent determination of its spectroscopic and catalytic properties. Before measurements, samples are kept at room temperature for 6 hours to ensure achieving thermodynamic equilibrium.

**Figure 5:**
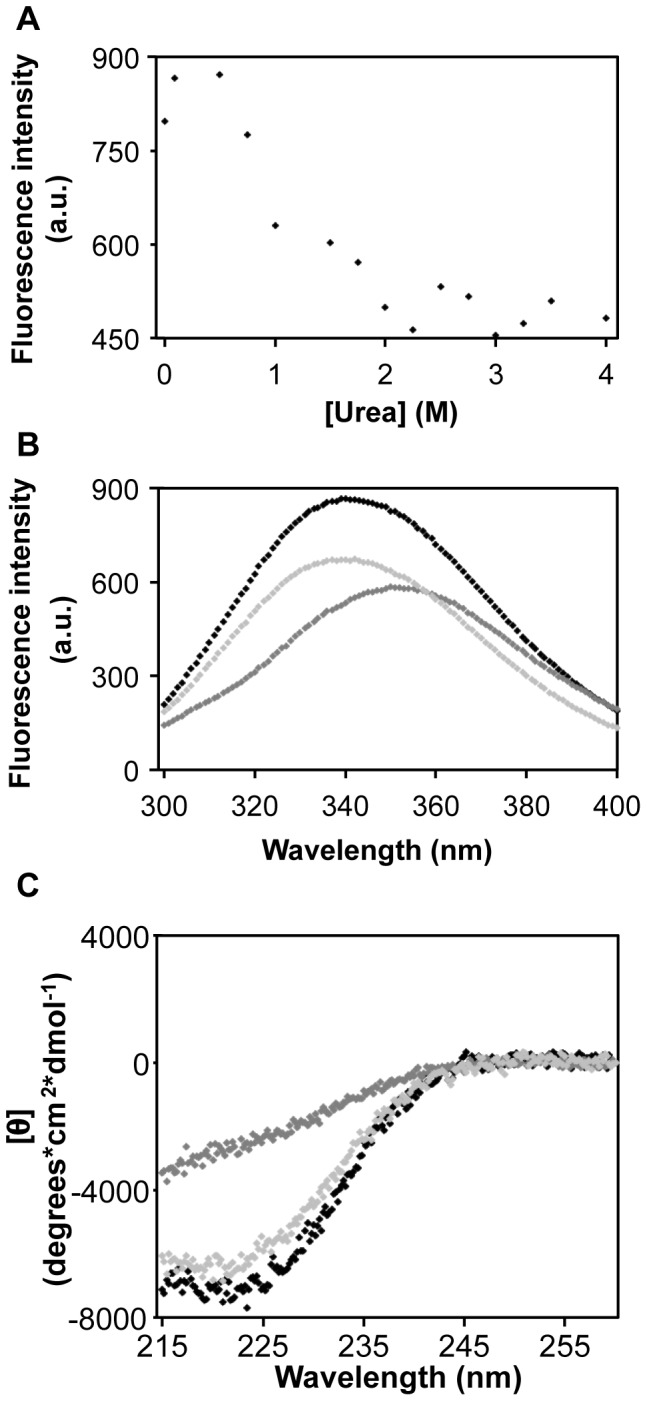
Denaturant-dependent folding of SERK1-KD. (**A**) Urea induced unfolding of SERK1-KD followed by fluorescence spectroscopy. Fluorescence intensity of 0.24 µM of SERK1-KD is measured at 340 nm, upon excitation at 280 nm. The native baseline ranges from 0 to about 0.5 M urea. Above 2 M urea, the unfolded baseline commences. The transition region of urea-induced protein unfolding ranges from about 0.5 to 2.0 M. (**B**) Fluorescence emission of native, unfolded and of refolded SERK1-KD. Black: native SERK1-KD in 0.09 M urea. Dark grey: unfolded SERK1-KD in 6 M urea. Light grey: refolded SERK1-KD, obtained by unfolding the protein for a period of 6 hours in 4.5 M urea, and subsequent dilution of denaturant to 0.09 M urea. Refolding took place during a period of 30 min at room temperature. Refolded SER1-KD has a fluorescence maximum at 340 nm, indicative of folded protein. Approximately 80% of unfolded SERK1-KD properly refolds, according to the difference in fluorescence intensity between native and refolded protein. (**C**) Far-UV CD spectra of native, unfolded and of refolded SERK1-KD. Black: native SERK1-KD in 0.45 M urea. Dark grey: unfolded SERK1-KD in 4.5 M urea. Light grey: refolded SERK1-KD, obtained by unfolding the protein for a period of 6 hours in 4.5 M urea, and subsequent dilution of denaturant to 0.45 M urea. Refolding took place during a period of 30 min at room temperature.

Upon urea-induced unfolding of SERK1-KD fluorescence at 340 nm decreases (Fig. 5a) and the maximum of fluorescence emission shifts from 340 to 350 nm (Fig. 5b). Up to urea concentrations of about 0.5 M, fluorescence intensity of the sample is large, indicating that this region of the unfolding curve of SERK1-KD is the native baseline (Fig. 5a). In this denaturant range, virtually all SERK1-KD molecules are in the native state. Upon increasing the denaturant concentration, the ratio of molecules in the native state to those in the unfolded state drops, and as a result fluorescence emission decreases. This decrease in fluorescence highlights the transition region of urea-induced SER1-KD unfolding, which ranges from about 0.5 to 2.0 M urea. Above 2 M urea, SERK1-KD molecules are in the unfolded state, giving rise to the unfolded baseline in the fluorescence unfolding curve of Fig. 5a.

**Figure 6:**
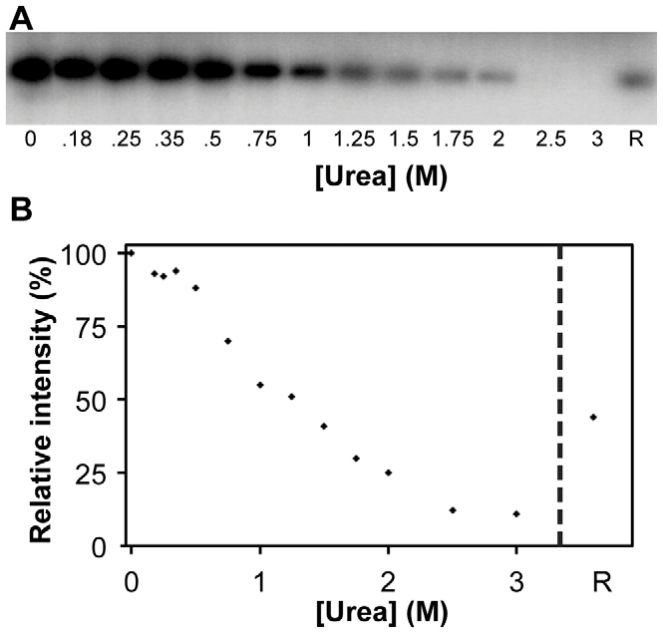
Dependence of SERK1-KD kinase activity on urea concentration. (**A**) Autoradiograph of autophosphorylation activity of SERK1-KD at different concentrations of urea. R: Refolded SERK1-KD, obtained by unfolding the protein for a period of 2 hours in 3 M urea and subsequent dilution of denaturant to 0.18 M urea. Refolding took place during a period of 30 min at room temperature. (**B**) Urea-dependent loss of phosphorylation activity of SERK1-KD. The gel presented in (A) is analysed with ImageJ to quantify intensities of the bands observed, which correlates with incorporation of radioactive phosphate. Loss of kinase activity happens gradually upon increasing urea concentration above 0.5 M. Phosphorylation activity is completely lost at concentrations urea above 2 M.

To assess whether diminishing of the population of the native state during urea induced unfolding is linked to loss of phosphorylation activity of SERK1-KD, protein activity is measured at increasing denaturant concentrations (Fig. 6). Up to 0.5 M urea, autophosphorylation activity only marginally decreases, most likely because all SERK1-KD molecules are in the native state. Upon increasing urea concentration above 0.5 M, loss of kinase activity happens gradually (Fig. 6). This denaturant-dependent loss of activity coincides with the observed diminished population of the native state of SERK1-KD, as reported by fluorescence emission (Fig. 5a). Above 2 M urea no incorporation of radioactive phosphate is observed, because SERK1-KD is unfolded and thus inactive.

The above data show that unfolding of SERK1-KD is intimately linked to loss of its phosphorylation activity.

### Upon spontaneous refolding in vitro, SERK1-KD regains native structure and catalytic activity

To investigate whether urea-induced unfolded SERK1-KD is able to spontaneously refold *in vitro*, the protein is first unfolded at high concentration denaturant (i.e., above 2 M urea, Fig. 5a). To induce refolding, urea concentration is then lowered to a value at which SERK1-KD molecules are in the native state (i.e., below 0.5 M urea (Fig. 5a)). Subsequently, we determine spectroscopic and catalytic properties of refolded SERK1-KD, to probe whether refolded protein has native three-dimensional structure.

Fluorescence emission of refolded SERK1-KD has its maximum at 340 nm, typical for native SERK1-KD (Fig. 5b). Approximately 80% of unfolded protein refolds properly, as comparison of fluorescence intensity of native and refolded protein indicates. The remaining fraction is most likely aggregated. Far-UV CD confirms that the large majority of unfolded SERK1-KD spontaneously refolds *in vitro* to native protein (Fig. 5c) because CD spectra of native and refolded SERK1-KD are similar. Again, due to some aggregation, the amplitude of the CD signal of refolded protein is slightly less than that of native protein. To evaluate whether refolded SERK1-KD also regains catalytic activity, incorporation of radioactive phosphate is probed. Figure 6 shows that refolded SERK1-KD indeed autophosphorylates, which is the hallmark for properly folded, catalytically active SERK1-KD. Compared to native SERK1-KD about 50% of autophosphorylation activity is recovered.

Protein concentration in the initial refolding sample (i.e., before dilution of denaturant) is an important parameter determining refolding efficiency, since aggregation highly depends on protein concentration. This concentration is highest in the refolding sample used for activity determination, explaining the lower refolding efficiency observed (Fig. 6) compared to refolding detected by spectroscopic techniques (Figs. 5b, c). Nevertheless, we conclude that fully active SERK1-KD can be obtained in the absence of any cellular context.

## Discussion

In this work we addressed whether the catalytic activity of LRR-RLK SERK1 from *Arabidopsis thaliana* is an inherent property of its kinase domain. Our results show that SERK1-KD regains its catalytic activity upon refolding *in vitro*, indicating that the protein has the intrinsic capacity to become active. Because *in planta* SERK1 RLK is a non-ligand binding co-receptor functioning in several signalling pathways, maintaining the inactive status of the kinase domain is necessary to prevent unscheduled signalling. Inactivation of the kinase domain could be achieved by either intra- or intermolecular inhibitory elements. One way by which such an inhibitory element could act is by maintaining parts of the protein disordered until activity is required.

The bioinformatics tool PONDR® VL-XT predicts that SERK1-KD is largely structured, with one extended region of disorder. This disordered region comprises part of its C-terminal tail and 41 amino acids in domains X and XI, regions previously identified as essential for both *in vitro* kinase phosphorylation activity and *in planta* function of SERK1 [15]. Disorder analysis of six other plant kinases indicates that the C-terminal tail of plant kinases functioning in signalling often is disordered. The C-terminal tail of BRI1 has been identified as an auto-inhibitory element for BRI kinase activity [50]. This could indicate that the C-terminal tail is a more common regulatory element in plant kinases. An extended region of disorder (i.e. more than 40 residues) in the catalytic kinase domain was only predicted for FLS2 and SERK1. Such a region can function as another form of kinase regulation, as was previously identified for epidermal growth factor receptor (EGFR). Molecular dynamics studies identified a region of disorder in the kinase domain of EGFR. This region is shown to be important for dimerization between two kinase domains after which it becomes more ordered [51]. SERK1 can form homo- and hetero-oligomers *in planta* [22]. While it has been proven that EGFR only functions as a dimer, it is not clear whether homodimerization plays a role in controlling the catalytic activity of SERK1, because purified SERK1-KD, which is monomeric according to analytical gelfiltration, is already catalytic active. The predicted disordered regions of SERK1 might have a function in interaction with ligand-perceiving RLK's with which members of the SERK family form hetero-oligomers [52].

Phosphorylation sites are often found in disordered regions of proteins, probably due to easy access of these sites [28]. Phosphorylation can induce structuring in disordered regions [53], [54], [55], or hardly influences these regions [56], [57]. In addition, phosphorylation can change the ability of these regions to undergo protein-protein interactions [58]. The number of phosphorylation sites differs vastly between SERK1 and its close paralogs SERK2 and BAK1 (SERK3) [14]. Comparison of previously identified phosphorylation sites of SERK1 and predicted disorder indicates that there is no bias towards phosphorylating disordered regions. More strikingly, seven of eight phosphorylation sites identified *in planta* are in predicted structured parts of the protein. Four of these sites are found in the activation loop of SERK1-KD (Thr459, Thr462, Thr463 and Thr468), of which phosphorylation is known to be important for kinase activity [7].

Fluorescence, far-UV CD and activity data presented in this paper show that SERK1-KD is largely structured and active *in vitro*. The observed coincidence of loss of activity and three-dimensional protein structure upon denaturant induced unfolding demonstrates that properly folded structure is essential for SERK1-KD to be active. Upon lowering denaturant concentration, unfolded SERK1-KD is able to refold to its native structure and regains catalytic activity. Thus, SERK1-KD folds to active kinase, while its cellular context comprising chaperones, membrane components and interaction partners, is absent. However, to counteract aggregation and make folding more efficient in the hugely crowded cellular context [59], chaperones most likely assist in folding SERK1-KD molecules *in vivo*.

For organisms it is important to keep RLK's inactive until their signalling is required to properly respond to signals. For plant RLK's this issue has not received much attention. We observe that SERK1-KD autonomously folds to its active state within 30 minutes. This observation suggests that *in planta* SERK1 already contains an active kinase domain before it is transported to its proper location, the plasma membrane [23], or incorporated in the correct protein complex required for signalling activity. Thus, mechanisms are likely needed *in planta* to keep kinase activity of SERK1 in check. For other plant RLK's candidate inhibitory proteins have indeed been identified, such as BKI1 or protein phosphate 2A for BRI1 [3], [60]. Dephosphorylation of specific residues essential for phosphorylation activity has also been proposed to deactivate RLK's [61]. A possible candidate inhibitor of SERK1 is KAPP, the PP2C protein phosphatase [23]. Finally, endoplasmatic reticulum associated protein degradation (ERAD) potentially plays a role during incorporation of SERK1-KD into correctly assembled receptor complexes, because ERAD also is important during biogenesis and quality control of other plant receptor complexes [62], [63], [64]. The next challenge is to elucidate whether and how the above mechanisms contribute to keeping SERK1-KD inactive *in planta*, until its activity is required.

## Supporting Information

Figure S1 **SDS-PAGE and Western blot of SERK1-KD.** Left: SDS-PAGE of aliquots taken at different steps during SERK1-KD purification. Lane 1, cell extract from BL21* *E. coli* cells expressing 6xHIS-SERK1-KD; lane 2; eluate from HIS-pure cobalt column; lane 3, eluate from SourceQ-15; lane 4, eluate from Superdex 75 10/300 GL (i.e., final, purified sample); lane 5, Marker. Right: Western blot of final purified protein, using anti- His-tag antibodies. Lane 1, purified SERK1-KD; lane 2, Marker.
(TIFF)

Figure S2 **SERK1-KD phosphorylation properties.** Left: Autophosphorylation of SERK1-KD (1 µg); Right: Transphosphorylation of casein (1 µg) by SERK1-KD (0.2 µg). Aliquots are taken at the time points indicated (in minutes) and subsequently separated by SDS-PAGE. Incorporation of ^32^P-yATP is visualized using a PhosphoImager.
(TIF)

Figure S3 **Phosphorylation status of native and autophosphorylated SERK1-KD.** Anti- Phosphoserine, -threonine and -tyrosine antibodies are used to probe the phosphorylation status of SERK1-KD. Lane 1, 0.75 µg of SERK1-KD after 45 min of incubation with ATP; lane 2, 0.75 µg of SERK1-KD as purified from *E. coli*; M, marker.
(TIF)

Figure S4 **Far-UV CD spectrum of refolded SERK1-KD.** SERK1-KD was first heated to 85°C and subsequently cooled to 15°C. The far-UV CD spectrum of this protein differs from the corresponding spectrum of native SERK1-KD shown in Fig. 2, and consequently thermally unfolded SERK1-KD does not properly refold upon lowering temperature.
(TIF)
