# Supplementary figures and images for: The Arabidopsis thaliana SERK1 Kinase Domain Spontaneously Refolds to an Active State In Vitro
Source: PLoS One. 2012 Dec 7;7(12):e50907. doi: 10.1371/journal.pone.0050907 (PMC3517577; doi:10.1371/journal.pone.0050907)

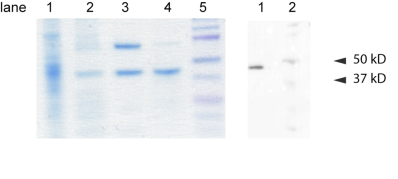

Supplement: Figure S1 — SDS-PAGE and Western blot of SERK1-KD. Left: SDS-PAGE of aliquots taken at different steps during SERK1-KD purification. Lane 1, cell extract from BL21* E. coli cells expressing 6xHIS-SERK1-KD; lane 2; eluate from HIS-pure cobalt column; lane 3, eluate from SourceQ-15; lane 4, eluate from Superdex 75 10/300 GL (i.e., final, purified sample); lane 5, Marker. Right: Western blot of final purified protein, using anti- His-tag antibodies. Lane 1, purified SERK1-KD; lane 2, Marker. (TIFF) [file pone.0050907.s001.tiff]

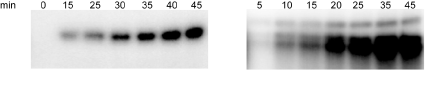

Supplement: Figure S2 — SERK1-KD phosphorylation properties. Left: Autophosphorylation of SERK1-KD (1 µg); Right: Transphosphorylation of casein (1 µg) by SERK1-KD (0.2 µg). Aliquots are taken at the time points indicated (in minutes) and subsequently separated by SDS-PAGE. Incorporation of 32P-yATP is visualized using a PhosphoImager. (TIF) [file pone.0050907.s002.tif]

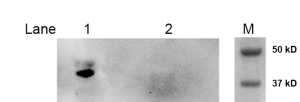

Supplement: Figure S3 — Phosphorylation status of native and autophosphorylated SERK1-KD. Anti- Phosphoserine, -threonine and -tyrosine antibodies are used to probe the phosphorylation status of SERK1-KD. Lane 1, 0.75 µg of SERK1-KD after 45 min of incubation with ATP; lane 2, 0.75 µg of SERK1-KD as purified from E. coli; M, marker. (TIF) [file pone.0050907.s003.tif]

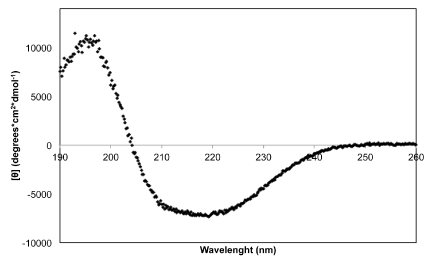

Supplement: Figure S4 — Far-UV CD spectrum of refolded SERK1-KD. SERK1-KD was first heated to 85°C and subsequently cooled to 15°C. The far-UV CD spectrum of this protein differs from the corresponding spectrum of native SERK1-KD shown in Fig. 2, and consequently thermally unfolded SERK1-KD does not properly refold upon lowering temperature. (TIF) [file pone.0050907.s004.tif]
